# Usefulness Differs Between the Visual Assessment and Specific Binding Ratio of ^123^I-Ioflupane SPECT in Assessing Clinical Symptoms of Drug-Naïve Parkinson’s Disease Patients

**DOI:** 10.3389/fnagi.2018.00412

**Published:** 2018-12-20

**Authors:** Hidetomo Murakami, Atsushi Kimura, Taro Yasumoto, Ayako Miki, Ken Yamamoto, Naohito Ito, Yutaro Momma, Yoshiyuki Owan, Satoshi Yano, Kenjiro Ono

**Affiliations:** Department of Neurology, School of Medicine, Showa University, Tokyo, Japan

**Keywords:** Parkinson’s disease, ^123^I-ioflupane SPECT, visual assessment, specific binding ratio, cognitive function, postural instability

## Abstract

**Background:** In clinical practice, assessment of the striatal accumulation in ^123^I-ioflupane single photon emission computed tomography (SPECT) is commonly performed calculating the specific binding ratio (SBR) for the whole striatum. On the other hand, visual assessment of striatal accumulation in the SPECT was recently established. However, correlations of visual assessment with motor and cognitive functions in Parkinson’s disease (PD) have rarely been examined. Differences in the usefulness of these assessments at clinics are uncertain.

**Objective:** We performed this study to compare correlations of cognitive and motor functions in drug-naive PD between the SBR and visual assessment using ^123^I-ioflupane SPECT.

**Methods:** Cognitive and motor assessments and ^123^I-ioflupane SPECT were performed in 47 drug-naïve PD patients with Mini-mental State Examination scores of ≥25. Cognitive function was assessed using the total score and 6 subscores of the Montreal Cognitive Assessment (MoCA) and 10 separate subtests of the Neurobehavioral Cognitive Status Examination (COGNISTAT). Motor function was assessed using the Hoehn and Yahr scale and Unified Parkinson’s Disease Rating Scale. Accumulation of ^123^I-ioflupane was determined by visual assessment based on five grades: 1, burst striatum; 2, egg-shaped; 3, mixed type; 4, eagle wing; 5, normal striatum; and by calculating SBR averaged for the bilateral striatum using the DaTView computer software commonly used in clinical practice. Each SPECT assessment was compared with each subscore for cognitive and motor assessments.

**Results:** Spearman correlation analysis showed SBR was significantly correlated with the MoCA subscores of visuospatial function and attention, and with COGNISTAT subtests of attention. Visual assessment showed significant negative correlation with the Hoehn and Yahr scale. Mean score of postural instability in patients with visual grade of 1 was significantly higher than those in patients with visual grades of 2 and 3.

**Conclusion:** Clinical symptoms reflected by ^123^I-ioflupane SPECT differ between the SBR and visual assessment. SBR reflects some cognitive functions, whereas a visual assessment grade of 1, which signifies decreased uptake of ^123^I-Ioflupane in the caudate nucleus, reflects postural instability. Thus, the caudate nucleus may play an important role in posture maintenance. Our results suggest that performing both assessments is of value.

## Introduction

^123^I-ioflupane single photon emission-computed tomography (SPECT) can detect the presynaptic dopamine transporter and has been introduced in clinical practice for differential diagnosis of Parkinson’s Disease (PD) and related disorders. Quantitative assessment of imaging, such as the ratio of specific-to-nonspecific uptake for the caudate nucleus and putamen, was used in previous research using the ^123^I-ioflupane SPECT. On the other hand, PD patients often show cognitive impairment. Among the cognitive domains, frontal lobe functions such as executive function are shown to correlate with uptake in the caudate (Nobili et al., 2010; Pellecchia et al., 2015) and putamen (Pellecchia et al., 2015) on ^123^I-ioflupane SPECT. However, assessment of the SPECT is performed by calculating the specific binding ratio (SBR) for the whole striatum using software such as DaTView (AZE Corp., Tokyo, Japan) in most clinics. This method was introduced by Tossici-Bolt et al. (2006). Analysis using this method is reported to be less dependent of partial volume effects, which would improve the consistency in quantitative measurements among different imaging devices. The multicenter database of healthy control for the SBR calculated by this method has been established in Japan (Matsuda et al., 2018). On the other hand, visual assessment using ^123^I-ioflupane SPECT has also recently been established (Eggers et al., 2011; Kahraman et al., 2012a,b). This classification is based on shape of lesions with uptake of ^123^I-ioflupane in parts of the striatum. Visual assessment is useful for distinguishing the motor subtype of PD (Eggers et al., 2011) and for differentiating PD from atypical parkinsonism (Kahraman et al., 2012b). However, the association of visual assessment with motor and cognitive functions has rarely been examined. Here, we compared association for some cognitive and motor functions between SBR for the whole striatum and visual assessment using ^123^I-Ioflupane SPECT.

## Materials and Methods

### Subjects

The subjects were 48 drug-naïve PD patients recruited from outpatient and inpatient groups diagnosed at Showa University Hospital and Showa University East Hospital, Tokyo, Japan. The diagnosis of PD was made using the United Kingdom Parkinson’s Disease Society Brain Bank criteria (Gibb and Lees, 1988). Patients were first diagnosed by clinical history and neurological findings before medication. Data of clinical and radiological assessments for this study were obtained within 2 months before medication. Then motor symptoms of all patients improved taking dopaminergic medication later, which confirmed the diagnosis of PD. MRI findings of all participants presented no abnormal intensity area nor focal atrophy suggesting parkinsonism other than PD. To exclude patients with apparent dementia, only those with a Mini-Mental State Examination (MMSE) score ≥25 were included. No subject had evidence of core clinical features of dementia with Lewy bodies (DLBs), such as fluctuating cognition with pronounced variations in attention and alertness, and recurrent visual hallucinations that are typically well formed and detailed. None were taking anti-Parkinsonian drugs, anti-dementia drugs, such as acetylcholinesterase inhibitors or *N*-methyl-D-aspartic acid (NMDA) receptor antagonists, or any drug that might influence striatum binding of ^123^I-ioflupane. No subjects had a disease other than PD that affected motor and cognitive functions. The Ethics Committee of Showa University School of medicine approved this study, and it was performed according to the Declaration of Helsinki. Written informed consent was obtained from all subjects.

### Assessment of Cognitive and Motor Functions

Cognitive function was assessed with the Montreal Cognitive Assessment (MoCA) and the Neurobehavioral Cognitive Status Examination (COGNISTAT). These neuropsychological tests were performed by examiners who were blinded to the patient’s motor assessment and radiological findings. We have shown that the MoCA and COGNISTAT detect cognitive impairment in PD patients sensitively, and are useful for detection of subtle cognitive impairment in PD without apparent dementia (Murakami et al., 2013). The MoCA was performed using the total score and 6 subscores as shown in Table 1. This categorization is the “domains-based subscore” presented in the original MoCA proposal (Nasreddine et al., 2005). One patient had a MoCA total score of 11, which signifies apparent dementia. Therefore, we excluded this case from the study. The other 47 patients were included in the study. The COGNISTAT has 10 separate cognitive examination subtests: orientation, attention, language-comprehension, language-repetition, language-naming, construction, memory, calculation, similarity, and judgment (Kiernan et al., 1987). Each subtest assesses different cognitive domains. In the Japanese version of the COGNISTAT, the raw score of each subtest is converted to a standard score, in which average scores in normal controls are set to 10 and standard deviations (SDs) in healthy controls are set to 1 (Matsuda et al., 2003). For cognitive assessment using the COGNISTAT, we used the standardized score of each subtest. In one case, raw score of attention subtest was too low and the standardized score for this case was not available. Therefore, we excluded this case for analysis using COGNISTAT attention subtest.

**Table 1:** Test items inclueded in the six MoCA subtests and motor subscores.

Battery	Subtest	Included items
MoCA	Visuospatial function	Copy cube, draw clock
	Attention	Digit span, vigilance, serial 7s
	Language	Naming of three animals, sentence repetition
	Executive function	Trail making, verbal fluency, abstraction
	Memory	Delayed recall of five words
	Orientation	Orientation
UPDRS	Tremor	Items 16, 20, and 21
	Rigidity	Item 22
	Akinesia	Items 23–26 and 31
	Gait	Items 13–15 and 29
	Postural instability	Items 27, 28, and 30


*MoCA, montreal cognitive assessment; UPDRS, unified Parkinson’s diesase rating scale.*

The Unified Parkinson’s Disease Rating Scale (UPDRS) (Movement Disorder Society Task Force on Rating Scales for Parkinson’s Disease, 2003) was used for assessment of motor function. This scale was assessed using the total score for Part III (motor score) and subscores for tremor, rigidity, akinesia, gait, and postural instability as shown in Table 1. All raw data for this study are included in the Supplementary Table S1.

### Imaging

^123^I-Ioflupane SPECT was performed using a standard protocol before medication with dopaminergic drugs. A dose of 167 MBq of ^123^I-ioflupane was injected intravenously at 10:20 to 10:30 a.m. Scanning was performed 3 h after the injection using a triple head gamma camera (GCA-9300R, Toshiba Medical Systems, Tochigi, Japan) equipped with low energy-high resolution fan-beam collimators (system resolution at 13.2 cm = 9.6 mm). A total of 128 projections for each detector were acquired on a 128 × 128 matrix (1.7 mm pixel size) over a circular 360 orbit. A 20% window centered at 159 KeV was used. The radius of rotation was 13.2 cm for a total acquisition time of 28 min. Image reconstruction was performed by filtered back projection using a Butterworth pre-filter (cut-off 0.76 cycles/cm, order 8). Attenuation and scatter corrections were not performed in image reconstruction. DaTView software (AZE Corp., Tokyo, Japan) that is commonly used in clinical practice was used to calculate SBR. The region of interest (ROI) was set manually at the bilateral putamen and caudate using template of standard shape as shown by Tossici-Bolt et al. (2006). The dimension of the template is approximately 61 mm × 48 mm and ensures the inclusion of all striatal counts. This technique is shown to be reproducible and sensitive (Tossici-Bolt et al., 2006). The entire cerebrum except the ROI was used as the reference region with non-specific tracer binding. The SBR was calculated as follows:

SBR = [specific binding (caudate and putamen) - nonspecific binding]/nonspecific binding.

The average of the right and left SBR was used for analysis. In one case, SBR presented *negative* value of -0.60. Striatal uptake of ^123^I-ioflupane in this case almost disappeared and the effect on SBR of regions with no uptake such as ventricles included in the ROI was relatively strong. Therefore, this SBR does not correctly reflect accumulation in the striatum and we excluded this case for analysis using SBR.

**FIGURE 1:**
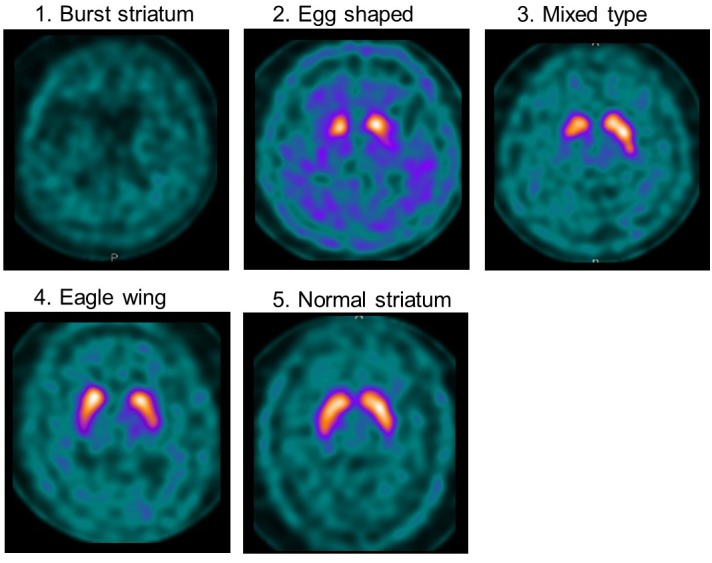
Examples of visual assessment using 123I-Ioflupane SPECT. Grade 1 (burst striatum): severe bilateral reduction with almost no uptake in both putamen and caudate nuclei or little uptake in the hemi-caudate nucleus with no uptake in the ipsilateral caudate nucleus. Striatal uptake of the same grade as background was judged as no uptake. Grade 2 (egg shaped type): bilateral reduction of tracer uptake with no uptake in putamen on either side and normal or almost normal uptake in caudate nuclei, resulting in an oval. Grade 3 (mixed type): normal or almost normal tracer uptake in bilateral caudate nuclei with asymmetrical tracer uptake in putamen. Grade 4 (eagle wing): normal bilateral uptake in caudate and almost normal and symmetrical uptake with a discrete reduction in one or both putamina, usually in lateral parts of the putamina and creating the shape of a wing. Grade 5 (normal striatum): symmetric tracer uptake bilaterally in putamen and caudate nuclei.

Visual assessment was performed using grades of 1–5, based on the shape of the lesion with ^123^I-ioflupane uptake on imaging as explained in Figure 1. Assessments of ^123^I-ioflupane were performed separately by two neurologists who were blinded to clinical symptoms. The assessments for 5 of 48 patients differed between the neurologists. Then they discussed with a third neurologist and the grade was determined.

### Statistical Analysis

Specific binding ratio and visual assessment of ^123^I-Ioflupane SPECT were compared with each subscore of the UPDRS, MoCA, and COGNISTAT. A Kolmogorov–Smirnov test showed that the visual assessment grades and subscores of all motor and cognitive assessments were non-normally distributed, and correlations between each assessment of ^123^I-ioflupane SPECT and each motor and cognitive assessment were then calculated using Spearman correlation analysis. To examine the influence of patient background factors, such as age, education, and duration from symptom onset, we performed multiple regression analyses using each cognitive assessment score showing significant correlation with SBR as the dependent variable and patient background factors as independent variables. Mean scores for motor and cognitive assessments that showed significant correlation with visual assessment were compared among visual grades using one-way analysis of variance (ANOVA, Tukey’s method for multiple comparison). No case had a visual grade of 5, and therefore this analysis was performed using subject groups with visual grades of 1–4. The level of significance was *p* < 0.05 (two-tailed probability).

## Results

**FIGURE 2:**
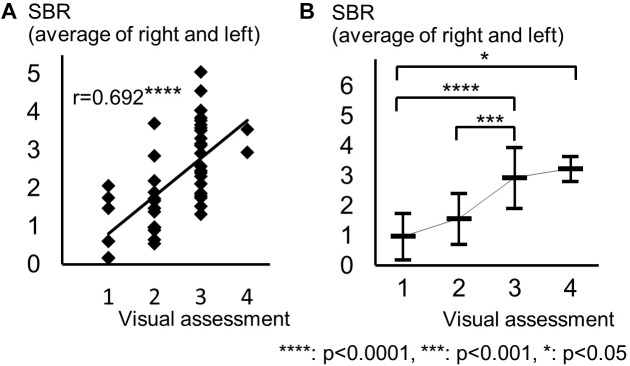
Distribution of each motor assessment score in each visual assessment grade. **(A)** Distribution of visual assessment grades and SBR in ^123^I-Ioflupane SPECT. **(B)** Mean and standard deviation of SBR in each visual assessment grade.

The background of the patients is shown in Table 2. The visual grades using ^123^I-ioflupane SPECT were 1, 2, 3, 4 and 5 in 8, 15, 22, 2 and 0 subjects, respectively. The visual grades were significantly positively correlated with SBR in ^123^I-ioflupane SPECT (*r* = 0.692, *p* < 0.0001; Figure 2A). Visual grade of one patient whose SBR was excluded from analysis was 1. SBRs (mean ± SD) were 0.98 ± 0.77, 1.57 ± 0.85, 2.94 ± 1.01, and 3.24 ± 0.42 for subjects with visual grades of 1, 2, 3, and 4, respectively, with significant differences (one-way ANOVA) in mean SBR between grades 1 and 3 (*p* < 0.0001), 2 and 3 (*p* < 0.001), and 1 and 4 (*p* < 0.05) (Figure 2B). However, subjects with SBR ≤ 2.06 had visual grades of 1, 2, and 3, and patients with SBR ≤ 3.70 had visual grade of 2 and 3 (Figure 2A).

**Table 2:** Patient background.

Male: Female	17:30
Age (years)	68.8 ± 12.8
Duration from symptom onset (years)	1.4 ± 1.2
Education (years)	13.4 ± 2.7
Hoehn and Yahr scale	2.3 ± 0.9
	(I: 10, II: 17, III: 18, IV: 1, V: 1 cases)
UPDRS part III (motor score)	17.1 ± 9.9
Onset symptom	Tremor: 21 cases
	Rigidity and/or akinesia: 26 cases
Visual assessment of SPECT	1: 8, 2: 15, 3: 22, 4: 2, 5: 0 cases


*UPDRS, unified Parkinson’s disease rating scale; SPECT, single photon emission computed tomography.*

**FIGURE 3:**
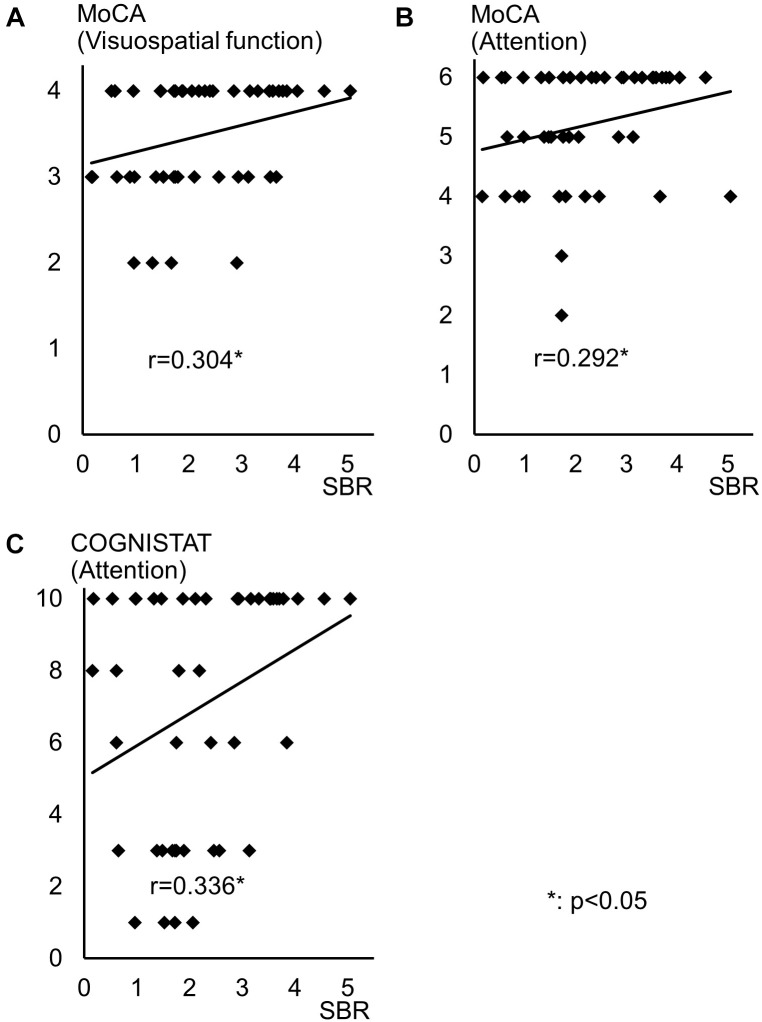
Distribution of SBR and scores for cognitive assessments with significant Spearman correlation coefficients. Distribution of SBR of ^123^I-Ioflupane SPECT with MoCA subscore of **(A)** visuospatial function and **(B)** attention, and **(C)** COGNISTAT attention subtest.

**FIGURE 4:**
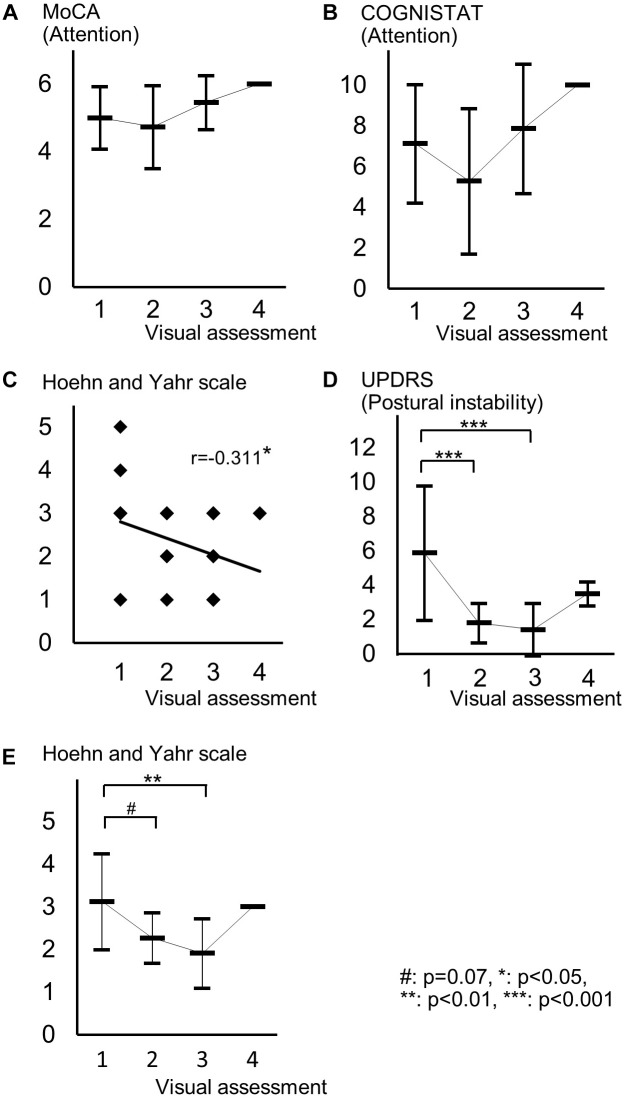
Distribution of cognitive and motor assessment scores for each visual assessment grade. **(A)** Mean and standard deviation of MoCA subscore of attention in each visual grade. **(B)** Mean and standard deviation of COGNISTAT subscore of attention in each visual grade. **(C)** Distribution of Hoehn and Yahr scale and visual grade. **(D)** Mean and standard deviation of postural instability score in each visual grade. **(E)** Mean and standard deviation of Hoehn and Yahr scale in each visual grade.

Spearman correlation coefficients for both SBR and visual assessments using ^123^I-ioflupane SPECT with each subscore for motor and cognitive assessments are shown in Table 3. Among cognitive assessments, SBR showed significant correlations with MoCA subscores for visuospatial function and attention and COGNISTAT subtest of attention (Table 3 and Figure 3). In multiple regression analyses, SBR was not independently correlated with any cognitive assessment (Table 4). There were independent correlations of education with MoCA visuospatial function subdomain; and duration from symptom onset with MoCA visuospatial function subtest (Table 4). Spearman correlation analysis showed that visual assessment correlated with MoCA and COGNISTAT subtests for attention (Table 3). However, mean scores for both subtests of attention showed no significant difference among visual grades (Figures 4A,B).

**Table 3:** Spearman correlation coefficients for relationships of SBR and visual assessment with motor and cognitive assessments.

		Assessment of ^123^I-ioflupane SPECT	
		
		SBR	Visual assessment
		
Battery	Subtest	*r*	*r*
MoCA	Total score	0.247	0.042
	Visuospatial function	0.304^*^	-0.083
	Attention	0.292^*^	0.322^*^
	Language	0.275	0.231
	Executive function	0.195	0.206
	Memory	0.091	-0.094
	Orientation	0.023	-0.009
COGNISTAT	Orientation	0.013	0.015
	Attention	0.336^*^	0.321^*^
	Language-comprehension	-0.038	-0.080
	Language-repetition	-0.001	-0.038
	Language-naming	0.021	0.046
	Construction	0.224	0.062
	Memory	0.112	-0.107
	Calculation	-0.040	0.014
	Similarity	0.158	-0.007
	Judgment	0.030	0.149
UPDRS	Part III	0.010	-0.150
	Tremor	-0.189	-0.209
	Rigidity	-0.020	-0.152
	Akinesia	0.205	0.061
	Gait	0.096	-0.083
	Postural instability	-0.121	*NA*
Hoehn and Yahr scale		-0.232	-0.311^*^


*SPECT, single photon emission computed tomography; SBR, specific binding ratio; MoCA, Montreal Cognitive Assessment; COGNISTAT, Neurobehavioral Cognitive Status Examination; UPDRS, Unified Parkinson’s Disease Rating Scale; NA, not available due to distribution of assessment scores. ^∗^*p* < 0.05.*

**Table 4:** Multiple regression analysis for cognitive assessments that had significant Spearman correlation coefficients with SBR.

		Independent variables			
		
		SBR	Age	Education	Duration from symptom onset

Battery	Subtest	*T*-value (*p*-value)	*T*-value (*p*-value)	*T*-value (*p*-value)	*T*-value (*p*-value)
MoCA	Visuospatial function	1.93	-1.10	2.35	2.95
		(0.061)	(0.276)	(0.023^∗^)	(0.005^∗∗^)
	Attention	0.47	-1.83	1.68	0.66
		(0.644)	(0.074)	(0.101)	(0.515)
COGNISTAT	Attention	1.35	-0.97	-0.03	1.24
		(0.186)	(0.337)	(0.974)	(0.223)


*SBR, specific binding ratio; MoCA, Montreal Cognitive Assessment; COGNISTAT, Neurobehavioral Cognitive Status Examination. ^∗^*p* < 0.05, ^∗∗^*p* < 0.01.*

For motor assessments, the visual assessment grade significantly correlated with Hoehn and Yahr scale (Table 3 and Figure 4C), whereas there was no significant correlation between SBR and any motor assessment score (Table 3). Postural instability scores (mean ± SD) were 5.88 ± 3.91, 1.80 ± 1.15, 1.41 ± 1.53, and 3.50 ± 0.71 for subjects with visual grades of 1, 2, 3, and 4, respectively, with significantly lower scores for grades 2 and 3 compared with grade 1 (both *p* < 0.001, one-way ANOVA) (Figure 4D). There was no significant difference in postural instability score between visual grades 2 and 3 (Figure 4D). Based on the distribution of these scores, calculating correlation coefficient between visual assessment and postural instability was inappropriate. Therefore, we did not perform this.

The Hoehn and Yahr scale scores (mean ± SD) were 3.13 ± 1.13, 2.27 ± 0.59, 1.91 ± 0.81, and 3.00 ± 0.00 for subjects with visual grades of 1, 2, 3, and 4, respectively, with significantly lower scores in patients with a visual grade of 3 compared with those with a grade of 1 (*p* < 0.01, one-way ANOVA) and a tendency for a difference in scores between grades of 1 and 2 (*p* = 0.07) (Figure 4E).

## Discussion

Some previous studies have shown that uptake of ^123^I-ioflupane in the caudate nucleus and/or putamen is significantly correlated with frontal/executive function in *de novo* PD patients (Nobili et al., 2010; Pellecchia et al., 2015). In our study, MoCA subscore of attention and the COGNISTAT subscore of attention were correlated with SBR. Performance of these subtests requires frontal/executive function such as attention and working memory. Therefore, our results agree with previous reports and suggest a link between the frontal lobe and striatum.

Age-related loss of the dopamine transporter and dopamine D2 receptor in the putamen and caudate has been shown (Ishibashi et al., 2009). SBR declines in an age-dependent manner (Matsuda et al., 2018) and age-related cognitive deficits are mediated by reduced dopamine transporter binding in the caudate and putamen (Erixon-Lindroth et al., 2005). These findings suggest that cognitive deterioration in PD is mediated by age-dependent deterioration in the striatum. However, our results using multiple regression analysis showed that education and disease duration, but not age, contribute to visuospatial function assessed by MoCA visuospatial function subtest. Many factors other than age are involved in cognitive deterioration. Therefore, our results showing SBR seemingly correlated with cognitive performance in drug-naïve PD signify SBR reflects some factors that affect cognitive impairment in PD. In clinical practice, SBR can be a reliable marker of cognitive function in drug-naïve PD patients without dementia. On the other hand, Spearman correlation analysis showed that MoCA and COGNISTAT subtests for attention correlate with visual assessment. However, the mean score for both subtests showed no significant difference among visual grades. Therefore, we believe that visual assessment does not reflect cognitive function.

In our study drug-naïve PD patients showed the mean score for postural instability for visual assessment grade 1 was significantly worse than that for grades 2 and 3 as shown in Figure 4D. There was no significant difference in postural instability score between visual grades 2 and 3. Visual grade 3 indicates uptake in the bilateral caudate nuclei and partial putamen, grade 2 shows uptake in only bilateral caudate nuclei, and grade 1 indicates no uptake in the striatum or little uptake in the hemicaudate nucleus. Therefore, our results suggest that patients with bilateral uptake in the caudate nuclei have better postural stability, suggesting the bilateral dopaminergic caudate nuclei plays an important role in posture maintenance. This is supported by a study by Rosenberg-Katz et al. showing that PD patients with fall had a lower volume in the caudate nuclei, but not in the putamen, compared with PD patients without fall and healthy controls (Rosenberg-Katz et al., 2015). PD patients with postural instability and gait disturbance also have decreased caudate activity (Zhang et al., 2016). The physiological mechanism of human upright posture is a product of a complex dynamic system that relies on integration of input from multimodal sensory sources, such as the visual, vestibular, and somatosensory systems, and higher cognitive function for assessment of postural stability (Slobounov et al., 2006). The caudate nucleus has functional networks with some areas in the cerebral cortex related to some cognitive functions (Zhang et al., 2016). Therefore, the caudate nucleus plays an important role in posture maintenance. Yoon and Lee (2014) examined the effect of balance and gait training on recovery of motor function in PD mouse models and showed both training improved motor function and increased expression of tyrosine hydroxylase, a marker of dopaminergic activity, in the striatum. This suggests that striatal function commanding posture and gait is dopaminergic. Therefore, our results support the physiological role of dopaminergic caudate nucleus for maintenancing posture.

In our subjects, the Hoehn and Yahr scale showed a significant correlation with the visual assessment grade and the mean score on this scale differed significantly between visual grades 1 and 3 and tended to differ between grades 1 and 2 as shown in Figure 4E. Therefore, in clinical practice, the visual assessment grade has a potential to be a marker of clinical stage on the Hoehn and Yahr scale.

Mean scores of postural instability and Hoehn and Yahr scale in patients with visual grade of 4 were higher than those in patients with visual grades of 2 and 3. This is because only two patients were included in the group of visual grade of 4. Our results should be ascertained by future studies using another cohort.

There are previous studies assessing striatal accumulation of ^123^I-ioflupane in the caudate nucleus and putamen, respectively (Nobili et al., 2010; Pellecchia et al., 2015). In clinical practice, SBR for the whole striatum, calculated by a commercially available computer software, such as DaTView, is commonly used in many clinics. Recently, a multicenter database of healthy control for the SBR calculated by this method has been established in Japan (Matsuda et al., 2018) and assessment of the imaging tends to depend on the SBR. However, our present study showed that SBR and visual assessment reflect different aspects of clinical symptoms in drug naïve PD patients. SBR overlapped among visul assessment grades as shown in Figure 2A. Subjects with SBR ≤ 2.06 had visual grades of 1, 2, and 3, and patients with SBR ≤ 3.70 had visual grades of 2 and 3. Our results show that visual assessment distinguished different aspects of PD patients with overlapped SBR. Therefore, performance of both assessments is recommended in clinical practice. Further studies in other cohorts are expected to be performed.

## Author Contributions

HM and YO performed the study design and concept, and statistical analysis. AK, TY, AM, KY, NI, YM, YO, and SY were involved in the acquisition of data. HM, YO, and KO performed the analysis and interpretation of data. HM performed the drafting of the manuscript. All the authors revised the manuscript critically for important intellectual content, contributed significantly to the latter version of the manuscript, and approved the final version on the manuscript.

## Conflict of Interest Statement

The authors declare that the research was conducted in the absence of any commercial or financial relationships that could be construed as a potential conflict of interest.

## Supplementary Material

TABLE S1 All raw data of participants included in this study.

## References

[B1] Eggers C. Kahraman D. Fink G. R. Schmidt M. Timmermann L. (2011). Akinetic-rigid and tremor-dominant Parkinson’s disease patients show different patterns of FP-CIT single photon emission computed tomography. *Mov. Disord.* 26 416–423. 10.1002/mds.23468 21264942

[B2] Erixon-Lindroth N. Farde L. Wahlin T. B. Sovago J. Halldin C. Bäckman L. (2005). The role of the striatal dopamine transporter in cognitive aging. *Psychiatry Res.* 138 1–12. 10.1016/j.pscychresns.2004.09.005 15708296

[B3] Gibb W. R. Lees A. J. (1988). The relevance of the Lewy body to the pathogenesis of idiopathic Parkinson’s disease. *J. Neurol. Neurosurg. Psychiatry* 51 745–752. 10.1136/jnnp.51.6.745 2841426 PMC1033142

[B4] Ishibashi K. Ishii K. Oda K. Kawasaki K. Mizusawa H. Ishiwata K. (2009). Regional analysis of age-related decline in dopamine transporters and dopamine D2-like receptors in human striatum. *Synapse* 63 282–290. 10.1002/syn.20603 19116949

[B5] Kahraman D. Eggers C. Holstein A. Schneider C. Pedrosa D. J. Dietlein M. (2012a). 123I-FP-CIT SPECT imaging of the dopaminergic state. Visual assessment of dopaminergic degeneration patterns reflects quantitative 2D operator-dependent and 3D operator-independent techniques. *Nuklearmedizin* 51 244–251. 10.3413/Nukmed-0449-11-12 22526237

[B6] Kahraman D. Eggers C. Schicha H. Timmermann L. Schmidt M. (2012b). Visual assessment of dopaminergic degeneration pattern in 123I-FP-CIT SPECT differentiates patients with atypical parkinsonian syndromes and idiopathic Parkinson’s disease. *J. Neurol.* 259 251–260. 10.1007/s00415-011-6163-1 21750954

[B7] Kiernan R. J. Mueller J. Langston J. W. Van Dyke C. (1987). The neurobehavioral cognitive status examination: a brief but differentiated approach to cognitive assessment. *Ann. Intern. Med.* 107 481–485. 10.1080/13607863.2012.702729 3631786

[B8] Matsuda H. Murata M. Mukai Y. Sako K. Ono H. Toyama H. (2018). Japanese multicenter database of healthy controls for [123I]FP-CIT SPECT. *Eur. J. Nucl. Med. Mol. Imaging* 45 1405–1416. 10.1007/s00259-018-3976-5 29478082 PMC5993845

[B9] Matsuda O. Kumazawa Y. Sakuraba Y. Matsuda H. Nakatani M. Saito M. (2003). The development of the Japanese version of the neurobehavioral cognitive status examination (NCSE), second report. *Jpn. J. Geriatr. psychiatry* 14 475–483.

[B10] Movement Disorder Society Task Force on Rating Scales for Parkinson’s Disease (2003). The unified parkinson’s disease rating scale (UPDRS): status and recommendations. *Mov. Disord.* 18 738–750. 10.1002/mds.10473 12815652

[B11] Murakami H. Fujita K. Futamura A. Sugimoto A. Kobayakawa M. Kezuka M. (2013). The montreal cognitive assessment and neurobehavioral cognitive status examination are useful for screening mild cognitive impairment in Japanese patients with Parkinson’s disease. *Neurol. Clin. Neurosci.* 1 103–108. 10.1111/j.2049-4173.2013.00032.x

[B12] Nasreddine Z. S. Phillips N. A. Bédirian V. Charbonneau S. Whitehead V. Collin I. (2005). The montreal cognitive assessment, MoCA: a brief screening tool for mild cognitive impairment. *J. Am. Geriatr. Soc.* 53 695–699. 10.1111/j.1532-5415.2005.53221.x 15817019

[B13] Nobili F. Campus C. Arnaldi D. De Carli F. Cabassi G. Brugnolo A. (2010). Cognitive-nigrostriatal relationships in de novo, drug-naïve Parkinson’s disease patients: a [I-123]FP-CIT SPECT study. *Mov. Disord.* 25 35–43. 10.1002/mds.22899 20058228

[B14] Pellecchia M. T. Picillo M. Santangelo G. Longo K. Moccia M. Erro R. (2015). Cognitive performances and DAT imaging in early Parkinson’s disease with mild cognitive impairment: a preliminary study. *Acta Neurol. Scand.* 131 275–281. 10.1111/ane.12365 25644029

[B15] Rosenberg-Katz K. Herman T. Jacob Y. Mirelman A. Giladi N. Hendler T. (2015). Fall risk is associated with amplified functional connectivity of the central executive network in patients with Parkinson’s disease. *J. Neurol.* 262 2448–2456. 10.1007/s00415-015-7865-6 26233691

[B16] Slobounov S. Wu T. Hallett M. (2006). Neural basis subserving the detection of postural instability: an fMRI study. *Motor Control* 10 69–89. 10.1123/mcj.10.1.69 16571908

[B17] Tossici-Bolt L. Hoffmann S. M. Kemp P. M. Mehta R. L. Fleming J. S. (2006). Quantification of [123I]FP-CIT SPECT brain images: an accurate technique for measurement of the specific binding ratio. *Eur. J. Nucl. Med. Mol. Imaging* 33 1491–1499. 10.1007/s00259-006-0155-x 16858570

[B18] Yoon Y. J. Lee B. H. (2014). Effects of balance and gait training on the recovery of the motor function in an animal model of Parkinson’s disease. *J. Phys. Ther. Sci.* 26 905–908. 10.1589/jpts.26.905 25013293 PMC4085218

[B19] Zhang L. Li T. N. Yuan Y. S. Jiang S. M. Tong Q. Wang M. (2016). The neural basis of postural instability gait disorder subtype of Parkinson’s disease: a PET and fMRI study. *CNS Neurosci. Ther.* 22 360–367. 10.1111/cns.12504 26842842 PMC6492838

